# A high density genetic map and QTL for agronomic and yield traits in Foxtail millet [*Setaria italica* (L.) P. Beauv.]

**DOI:** 10.1186/s12864-016-2628-z

**Published:** 2016-05-04

**Authors:** Xiaomei Fang, Kongjun Dong, Xiaoqin Wang, Tianpeng Liu, Jihong He, Ruiyu Ren, Lei Zhang, Rui Liu, Xueying Liu, Man Li, Mengzhu Huang, Zhengsheng Zhang, Tianyu Yang

**Affiliations:** Engineering Research Center of South Upland Agriculture, Ministry of Education, Southwest University, Chongqing, 400716 People’s Republic of China; Crop Research Institute, Gansu Academy of Agricultural Sciences, Lanzhou, 730070 Gansu People’s Republic of China

**Keywords:** Genetic map, QTL, Agronomic traits, Yield traits, Foxtail millet (*Setaria italica* L.)

## Abstract

**Background:**

Foxtail millet [*Setaria italica* (L.) P. Beauv.], a crop of historical importance in China, has been adopted as a model crop for studying C-4 photosynthesis, stress biology and biofuel traits. Construction of a high density genetic map and identification of stable quantitative trait loci (QTL) lay the foundation for marker-assisted selection for agronomic traits and yield improvement.

**Result:**

A total of 10598 SSR markers were developed according to the reference genome sequence of foxtail millet cultivar ‘Yugu1’. A total of 1013 SSR markers showing polymorphism between Yugu1 and Longgu7 were used to genotype 167 individuals from a Yugu1 × Longgu7 F_2_ population, and a high density genetic map was constructed. The genetic map contained 1035 loci and spanned 1318.8 cM with an average distance of 1.27 cM between adjacent markers. Based on agronomic and yield traits identified in 2 years, 29 QTL were identified for 11 traits with combined analysis and single environment analysis. These QTL explained from 7.0 to 14.3 % of phenotypic variation. Favorable QTL alleles for peduncle length originated from Longgu7 whereas favorable alleles for the other traits originated from Yugu1 except for qLMS6.1.

**Conclusions:**

New SSR markers, a high density genetic map and QTL identified for agronomic and yield traits lay the ground work for functional gene mapping, map-based cloning and marker-assisted selection in foxtail millet.

**Electronic supplementary material:**

The online version of this article (doi:10.1186/s12864-016-2628-z) contains supplementary material, which is available to authorized users.

## Background

Foxtail millet (*Setaria italica* L.) has a long history of cultivation in China. Archaeological evidence indicated that foxtail millet was cultivated in some sites near the Yellow River before ca. 5000–6000 BC [1]. Because grains of foxtail millet are enriched for various amino acids and nutritive minerals and the crop possesses some advantageous traits, e.g. high photosynthetic efficiency and drought tolerance, foxtail millet is still a very important crop in arid and semiarid regions of northern China [2].

Foxtail millet has a short generation time (depending on the accession, approximately 5–8 weeks from planting to flowering, 8–15 weeks from planting to seed maturity) and can produce hundreds of seeds per inflorescence [3]. Seeds of foxtail millet are generally not dormant and can easily be cultivated at density of up to 100 plants/m^2^ in the glasshouse or in the field in temperate or tropical regions [3]. Because of its small genome (∼515 Mb) with a small number of chromosomes (2n = 2x = 18) and inbreeding nature, foxtail millet is a valuable model for investigating plant architecture, drought tolerance and C_4_ photosynthesis of grain and bioenergy crops [3–6]. Therefore, development of high yielding, high quality, and stress resistant foxtail millet cultivars is an important goal for foxtail millet scientists.

A high-contiguity “reference” genome sequence provides a natural platform for unifying information from a range of sequence-tagged DNA marker systems, toward the efficient application of new approaches to build upon knowledge of the biology of an organism [7]. Most major crops now have a reference genome sequence, and some have projected that within a few years all of the ~200 widely used domesticates will have such a resource [8]. Zhang et al. [9] produced a draft genome (~423 Mb) for foxtail millet (*S. italica*) that was anchored onto nine chromosomes and included 38,801 annotated genes. Bennetzen et al. [10] generated a high-quality reference genome sequence, and the ~400-Mb assembly covered ~80 % of the genome and >95 % of the gene space. Genome information for foxtail millet provides an important resource for crop improvement. Based on the reference genome (https://phytozome.jgi.doe.gov/pz/portal.html), Pandey et al. [11] designed 21,294 microsatellite primer pairs, and a total of 15,573 markers were physically mapped on 9 chromosomes of foxtail millet. Jia et al. [12] identified 2.58 million SNPs and used 0.8 million common SNPs to construct a haplotype map of the foxtail millet genome. Zhang et al. [13] isolated 5020 highly repetitive microsatellite motifs and designed 733 SSR primer pairs, which could produce reproducible amplicons and were polymorphic among 28 *Setaria* genotypes. Yadav et al. [14] identified a total of 30.706 TEs and developed 20,278 TE-based markers.

Genetic mapping is an essential prerequisite for activities such as marker-assisted selection, gene/quantitative trait loci (QTL) cloning, genome sequence assembly, association mapping, and evolutionary studies [15]. For foxtail millet (*S. italica* L.), the first available genetic linkage map was constructed from a cross between cultivars Longgu 25 and Pagoda Flower Green, and the map included 160 RFLP markers spanning 964 cM [16]. Jia et al. [2] constructed an integrated map with 81 SSR and 20 RFLP markers using an F_2_ population from a cross between *S. italica* acc. B100 and *S. viridis* acc. A10. Zhang et al. [9] constructed a genetic map including 751 markers using a Zhanggu × A2 F_2_ population with 480 individuals. Bennetzen et al. [10] constructed an interspecific genetic map including 992 SNP markers and covering 1416 cM.

Virtually all yield component traits and most agronomic traits of foxtail millet are quantitative inheritance, so it takes much time to increase yield and improve quality through traditional genetic improvement methods. Based on QTL identified for quantitative traits, molecular marker-assisted selection can rapidly increase yield and improve quality of foxtail millet cultivars. Doust et al. [17] located 25 QTL for vegetative branching and inflorescence architecture and identified candidate genes for control of branching from a cross between *S. italica* acc. B100 and *S. viridis* acc. A10. Wang et al. [18] detected two QTL related to plant height, one related to panicle length, one related to panicle and one related to grain weight using a Shen3 × Jinggu20 F_2_ population. Sato et al. [19] mapped *stb1* more precisely on chromosome 2. Mauro-Herrera et al. [20] identified 16 flowering time QTL. Additional, Gupta et al. [21] identified eight SSR markers on different chromosomes showing significant association with nine agronomic traits through association mapping.

To date, a large number of foxtail millet SSR markers have been developed, but SSRs used to construct a genetic map are limited. Furthermore, the available foxtail millet genetic maps have a limited number of markers, and the QTL identified are far from the linked makers. Therefore, it is urgent to explore more SSR markers to construct high-density linkage maps and identify many more QTL for marker-assisted selection in foxtail millet.

In the present study, two foxtail millet cultivars Yugu1 and Longgu7 were crossed to establish an F_2_ population. SSR markers developed from the foxtail millet genome sequence were applied to construct genetic map and explore favorable QTL alleles from either parent to increase yield and optimize agronomic traits. The results will be valuable for future research on improvement of foxtail millet yield and agronomic traits.

## Result

### Phenotypic analysis of agronomic and yield traits

Phenotypic analysis of agronomic traits was summarized in Additional file 1: Table S1. All 11 agronomic and yield traits showed a wide range of variation in 2013 and 2014 (Additional file 2: Figure S1). Skewness and kurtosis tests showed that these traits had approximately normal distributions. Complex significant correlations exist among agronomic traits (Additional file 3: Table S2). Period of duration from sprout to mature was significantly negatively correlated with other traits, except 1000-grain weight, in 2013. In contrast, period of duration was positively correlated with other traits, except peduncle length and main panicle length, in 2014. The other traits have positive correlations with one another in 2013 and 2014, except that main panicle diameter had a non-significantly negative correlation with 1000-grain weight, and peduncle length was negatively correlated with diameter of main stem, node number of main stem, and main panicle diameter in 2014.

### Physical mapping of SSR markers in the foxtail millet genome

A total of 10598 SSR markers were developed from the ‘Yugu1’ reference genomic sequence (Additional file 4: Table S3 http://www.ncbi.nlm.nih.gov/probe/?term=JAK%5Bsubm%5D%20). Primer sequence alignment showed that the 10598 SSR markers are different from those previously reported by Pandey et al. [11] and Zhang et al. [13]. A total of 10535 SSR markers were located on 9 chromosomes, and the remaining 63 were located on unmapped scaffolds. The number of SSR markers on the chromosomes ranged from 658 to 1874, and the markers collectively covered approximately 99.89 % of the physical length of the genome (Table 1). The marker density along each chromosome ranged from 16.17 to 31.78 markers per Mb, with an average of 26.25 markers per Mb. The highest marker density per Mb (31.78/Mb) was on chromosome 9, followed by 30.80/Mb on chromosome 1, 30.24 Mb on chromosome 5, and the lowest marker density per Mb (16.17/Mb) was on chromosome 8.

**Table 1:** Number and coverage of SSR markers on the chromosomes of *S. italica* L

Chr.	SSR marker	Cover length (Mb)	Chr. length (Mb)	Coverage (%)	Density (marker/Mb)
1	1298	42.13	42.15	99.95	30.80
2	1307	49.20	49.20	99.99	26.56
3	1335	50.64	50.65	99.98	26.36
4	891	40.22	40.41	99.53	22.05
5	1429	47.23	47.25	99.96	30.24
6	827	36.00	36.02	99.96	22.96
7	916	35.94	35.96	99.93	25.47
8	658	40.68	40.69	99.97	16.17
9	1874	58.83	58.97	99.76	31.78
total	10535	400.87	401.30	99.89	26.25

The most abundant type of SSR was pentanucleotides (4780, 45.85 %), followed by hexanucleotides (2295, 22.01 %), tetranucleotides (1925, 18.47 %), trinucleotides (782, 7.5 %), dinucleotides (376, 3.6 %) and mononucleotides (267, 2.56 %). Biased distributions of SSR motifs were detected on all nine chromosomes. For example, more mononucleotide microsatellite fragments containing C & G units were isolated, and more pentanucleotide SSR containing AAAAG & GAAAA, TTTTC & CTTTT, AAAAT &ATTTT and TTTCT & TCTTT were isolated (Additional file 5: Figure S2).

**Fig. 1:**
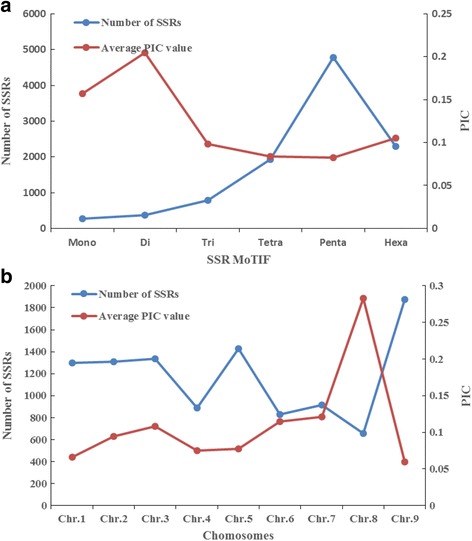
PIC variation among SSR motifs (**a**) and chromosomes (**b**)

Among the diverse SSR types developed in the present study, a higher level of genomic variants was detected among the ‘di-’ types (Fig. 1). The levels of SSR polymorphism and genomic variants were highest on foxtail millet chromosome 8 (Fig. 1). The PIC value for each chromosome ranged from 0.059 to 0.282, with a mean of 0.111. Among the diverse kinds of SSR motif, using the ‘di’ type as an example, AT & TA motif-containing markers gave the highest PIC value, while CG & GC motif-containing markers showed the lowest genetic diversity among the accessions sampled in this study.

### Genetic linkage map

Among 10598 SSR markers screened, 1013 (9.6 %) showed clear polymorphism between Yugu1 and Longgu7. The polymorphic markers were used to genotype the (Yugu1 × Longgu7) F_2_ population, and 1035 loci were produced. The 1035 loci were mapped into nine chromosomes, covering 1318.8 cM, with average distance of 1.27 cM between adjacent markers (Table 2).

**Table 2:** Distribution of loci and distorted loci in the Yugu1 x Longgu7 map

Chr.	Loci	Recombinant Length (cM)	Average interval (cM)	SD^a^ loci	SD ratio (%)
1	86	133.2	1.55	24	27.9
2	123	128.9	1.05	5	4.1
3	144	182.6	1.27	0	0.0
4	67	113.9	1.70	12	17.9
5	111	176.7	1.59	0	0.0
6	95	129.3	1.36	56	58.9
7	111	102.9	0.93	0	0.0
8	186	124.9	0.67	57	30.6
9	112	226.4	2.02	66	58.9
Total	1035	1318.8	1.27	220	21.3

^a^ Segregation Distortion

**Fig. 2:**
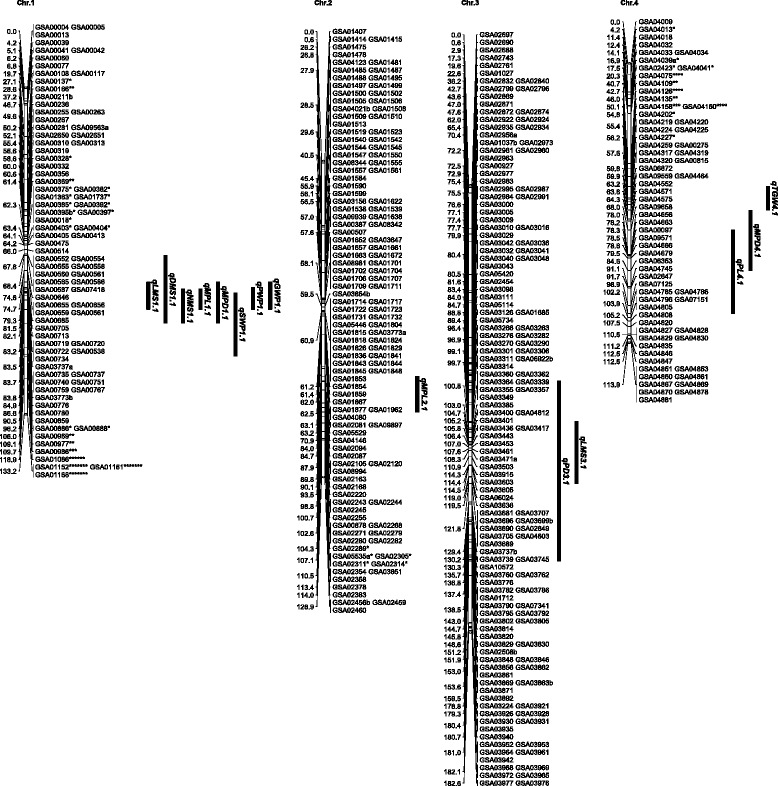
Genetic linkage map and QTL controlling agronomic and yield traits for Chr.1, Chr.2, Chr.3 and Chr.4. On each chromosome, the name of each marker is shown on the right. The number on the left indicates the genetic distance in cM. QTL were identified for 11 agronomic and yield traits and shown as period of duration (PD), peduncle length (PL), length of the main stem (LMS), diameter of the main stem (DMS), node number of the main stem (NMS), main panicle length (MPP), main panicle diameter (MPD), straw weight per plant (SWP), panicle weight per plant (PWP), grain weight per plant (GWP), and 1000-grain weight (TGW)

**Fig. 3:**
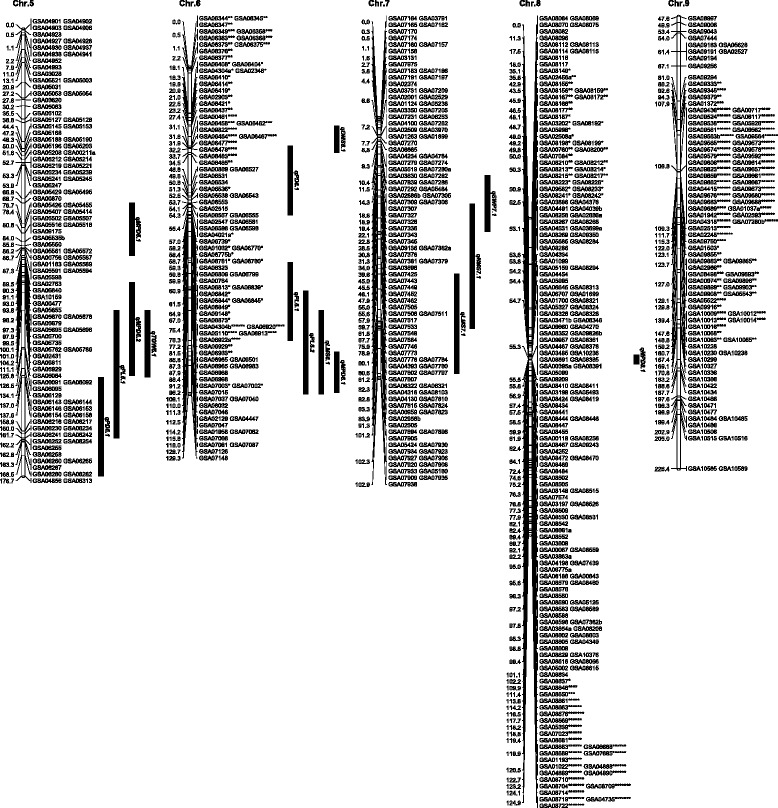
Genetic linkage map and QTL controlling agronomic and yield traits for Chr.5, Chr.6, Chr.7, Chr.8 and Chr.9

Loci were not evenly distributed over chromosomes. For example, Chr. 8 was mapped with 186 loci, whereas Chr. 1 and Chr. 4 were mapped with only 86 and 67 loci, respectively. The longest chromosome in terms of recombinational length was Chr. 9, which spanned 226.4 cM, and the shortest was Chr. 7, which spanned only 102.9 cM. Four large gaps (>20 cM) were identified on Chr. 2 (25.7 cM), Chr. 4 (20.4 cM), Chr. 5 (21.4 cM) and Chr. 9 (21.4 cM) (Figs. 2 and 3).

**Fig. 4:**
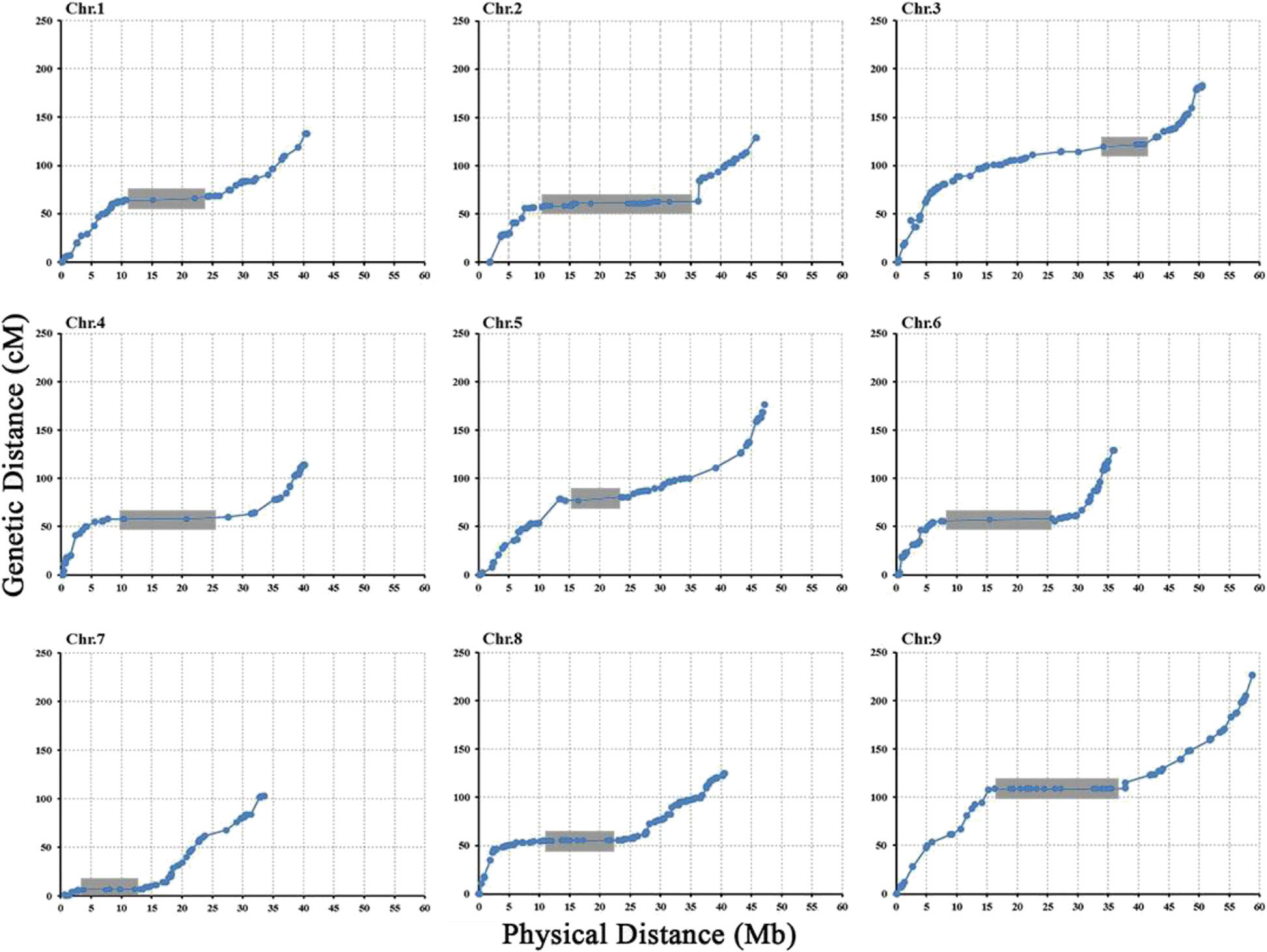
Genetic distance vs. physical distance for 1035 loci in foxtail millet. Genetic position of the 1035 polymorphism loci was plotted against the corresponding physical position. The shaded regions with very low recombination rates could be the centromere

To study colinearity and genome variations [10], dot plots were made comparing genetic maps and the reference genome sequence (Fig. 4). The average ratio of genetic-to-physical distance in low- and high-recombination chromosomes was 2.8 cM/Mb (Chr.2) and 3.85 cM/Mb (Chr.9), respectively (Table 3). All these SSR markers in the genetic map covered 395.65 Mb of physical length, which spanned about 76.8 % of the entire recombinational length of the foxtail millet genome (∼515 Mb).

**Table 3:** Genetic and physical distances among 1035 loci in foxtail millet

Chr.	SSR marker	Genetic distance(cM)	Physical distance(Mb)	Chr. length (Mb)	Average ratio of genetic-to-physical distance(cM/Mb)	Maker density (marker/Mb)
1	86	133.2	40.6	42.13	3.28	2.04
2	123	128.9	45.84	49.2	2.81	2.50
3	144	182.6	50.55	50.64	3.61	2.84
4	67	113.9	40.21	40.22	2.83	1.67
5	111	176.7	47.18	47.23	3.75	2.35
6	95	129.3	35.98	36	3.59	2.64
7	111	102.9	35.88	35.94	2.87	3.09
8	186	124.9	40.63	40.68	3.07	4.57
9	112	226.4	58.78	58.83	3.85	1.90
total	1035	1318.8	395.65	400.87	3.33	2.58

### Marker segregation distortion

Among the 1035 loci, 220 (21.3 %) showed segregation distortion (*P* < 0.05) with 107 (48.6 %) favoring Yugu1 alleles and 108 (49.1 %) favoring Longgu7 alleles (Table 2). The remaining distorted loci (5) favored the heterozygous genotype, and are clustered on Chr. 2. Distorted loci were unevenly mapped on different chromosomes. No distorted loci were mapped on Chrs. 3, 5 and 7, whereas 66 loci distorted toward Yugu1 were clustered on Chr. 9, and 12 and 56 loci distorted toward Longgu7 clustered on Chrs. 4 and 6, respectively. Chrs. 1 and 8 each had two large segregation distortion regions (SDR). There were 15 loci distorted toward Yugu1 and 9 distorted toward Longgu7 on Chr. 1. There were 26 loci distorted toward Yugu1 and 31 distorted toward Longgu7 on Chr. 8. Distorted loci toward the same allele appeared on the same chromosome or within the same SDR.

### QTL for agronomic and yield traits

A total of 29 QTL were identified for 11 agronomic and yield traits with a range of 1–6 QTL per trait (Table 4). The percentage of phenotypic variance explained by individual QTL for each trait ranged from 7.0 to 14.3 %. Among these QTL, 18 were detected from both combined analysis and single environment analysis and two were detected only from combined analysis. There were 22 favorable alleles originating from Yugu1 and 6 from Longgu7. For each QTL, the favorable allele originated from the same parent as indicated by the additive effect of QTL, except that qTGW5.1 were conferred by different parents in 2014 and combined.

**Table 4:** QTL controlling agronomic and yield traits in the Yugu1 × Longgu7 F2 population

Trait	QTL	Chr.	Year	Nearest marker	LOD	Additive	PVE (%)
PD (d)	qPD3.1	3	2014	GSA03737b	2.56	1.98	7
			Combined	GSA03029	2.66	0.56	7.3
	qPD5.1	5	2014	GSA06282	3.12	2.2	8.5
			Combined	GSA06282	3.59	1.55	9.7
	qPD6.1	6	Combined	GSA06527	2.64	1.22	7.2
PL (cm)	qPL5.1	5	2013	GSA06158	2.93	−2.34	8.1
	qPL6.1	6	2013	GSA06988	3.71	−2.22	10.1
			Combined	GSA07015	5.02	−2.35	13.3
	qPL6.2	6	2014	GSA07046	2.63	−2.2	7.1
	qPL4.1	4	2014	GSA04745	2.71	−1.58	7.4
LMS(cm)	qLMS1.1	1	2013	GSA00776	3.92	8.29	10.5
	qLMS7.1	7	2013	GSA07802	2.74	6.3	7.5
			Combined	GSA07802	3.04	4.4	8.3
	qLMS3.1	3	2014	GSA03915	2.58	5.6	7.1
			Combined	GSA03443	2.98	5.1	8.1
	qLMS6.1	6	2014	GSA07126	3.94	−6.24	10.6
			Combined	GSA07087	3.01	−4.9	8.2
DMS(cm)	qDMS1.1	1	2013	GSA00780	4.95	0.09	13.1
			Combined	GSA00780	4.85	0.06	12.9
	qDMS6.1	6	Combined	GSA06467	2.7	0.04	7.4
NMS(no)	qNMS1.1	1	2013	GSA00776	4.93	0.63	13.1
			Combined	GSA00780	3.6	0.56	9.7
	qNMS7.1	7	2013	GSA07533	2.9	0.5	7.9
MPL(cm)	qMPL1.1	1	2013	GSA00767	4.88	2.06	13
			Combined	GSA00767	4.49	1.24	12
	qMPL2.1	2	2014	GSA02220	4.19	1.6	11.2
			Combined	GSA02220	2.97	1.2	8.1
MPD(cm)	qMPD1.1	1	2013	GSA00767	3.84	0.22	10.3
			Combined	GSA00767	3.52	0.15	9.5
	qMPD4.1	4	2013	GSA04656	3.35	0.21	9.1
			Combined	GSA04656	2.75	0.13	7.5
	qMPD5.1	5	2013	GSA05516	3.67	0.22	9.9
			Combined	GSA05516	3.53	0.16	9.5
	qMPD5.2	5	2013	GSA06084	3.51	0.24	9..5
	qMPD8.1	8	2013	GSA08391	3.84	0.05	10.3
			Combined	GSA08391	2.99	0.05	8.1
	qMPD6.1	6	2014	GSA07087	2.7	0.02	7.4
SWP(g)	qSWP1.1	1	2013	GSA00780	5.45	3.43	14.3
			Combined	GSA00780	3.07	2.31	8.3
	qSWP7.1	7	2014	GSA07381a	2.8	1.27	7.6
PWP(g)	qPWP1.1	1	2013	GSA00767	4.72	3.26	12.6
			Combined	GSA00767	3.78	2.31	10.2
GWP(g)	qGWP1.1	1	2013	GSA00767	4.35	2.87	11.6
			Combined	GSA00767	3.25	1.99	8.8
TGW(g)	qTGW4.1	4	2013	GSA04227	2.9	−0.14	7.9
	qTGW5.1	5	2014	GSA06084	2.91	0.07	8
			Combined	GSA06092	2.94	−0.02	8

+ and−: Positive values indicate that the Yugu1 allele increased the trait value and negative values indicate that the Longgu7 allele increased the trait value

“Combined”: the average data of agronomic and yield traits from 2013 and 2014

Traits are period of duration (PD), peduncle length (PL), length of main stem (LMS), diameter of main stem (DMS), node number of main stem (NMS), main panicle length (MPP), main panicle diameter (MPD), straw weight per plant (SWP), panicle weight per plant (PWP), grain weight per plant (GWP), and 1000-grain weight (TGW)

## Discussion

### GSA primer pairs

Since the foxtail millet genome sequence has been determined, a large number of genome-wide versatile makers have been developed from the reference genome [9–14, 19]. However, a limited number of markers were applied to construct genetic linkage maps and identify QTL for agronomic and yield traits, except for 79 SSR detected by Sato et al. [19] and 0.8 million SNP detected by Jia et al. [12]. Although SNP markers have many advantages for genetic mapping and QTL mapping, SSR markers are still useful because of low cost and the use of standard equipment for genotyping large populations. Furthermore, SSRs have become a marker of choice in genotyping because of their high abundance, high level of allelic variation, co-dominant inheritance and analytical simplicity [13]. In this study, we have developed 10598 SSR markers based on the reference genome sequence. The newly developed markers were different from those previously developed by Zhang et al. [13] and Pandey et al. [11]. Among the 10598 SSR markers, 1013 showing polymorphism between Yugu1 and Longgu7 were applied to construct a genetic map and identify QTL for agronomic and yield traits. Therefore, the newly developed SSR markers in the present study were useful for fine-mapping, map-based cloning and molecular marker assisted breeding.

The data presented here support findings in several previous reports [2, 22, 23], that dinucleotide repeat unit microsatellites show higher levels of polymorphism in foxtail millet than other SSR motifs. However, in the present study, only a small portion of SSR (376, 3.6 %) were dinucleotide repeats. Additionally, the GC & CG dinucleotide repeats were few in number and showed lower levels of polymorphism in this study than other motifs, similar to the other reports [24–26].

### High-density genetic map

Genetic maps often have the problem of unevenly distributed markers, resulting in gaps. During meiosis, recombination does not occur evenly over the chromosomes. Further, marker sequences are not evenly, or even randomly dispersed, especially sequence repeats such as SSR [7]. The present genetic map contained 1035 loci, spanning 1318.8 cM, with an average of 1.27 cM between adjacent loci. Compared to other published foxtail millet interspecific genetic maps [2, 17, 20] and the intraspecific map [9, 16, 18, 19], the present map is the most saturated, mainly due to the large number of SSR primer pairs and approximately even distribution of loci across the genome.

The microsatellites were also not randomly or evenly distributed over the nine chromosomes of foxtail millet. For instance, chromosomes with fewer SSR loci (Chr. 1, Chr. 4) might have low marker diversity between the two parents of our population. The recombinational lengths of some chromosomes (e.g. Chr. 9) were much longer than others, similar to other studies [9, 16, 19].

### Segregation distortion

Segregation distortion may arise from lethality, partial male or female sterility, gametic selection or zygotic selection [27], and is common in mapping populations. Wang et al. [16] indicated severe segregation distortion on chromosome VIII, which suggested the presence of a gametocidal gene. In the present study, there were two SDRs at the middle-upper and bottom of Chr. 8. The distorted loci in two SDRs skewed toward different parents, suggesting that there may be two gametocidal genes (*Gc*) on Chr. 8. In wheat, gametocidal genes (*Gc*) in hetero- or hemizygous condition kill both male and female gametes lacking *Gc* genes [28]. On the other hand, 66 loci significantly distorted toward Yugu1 were clustered on Chr. 9, as reported by Sato et al. [19], suggesting that there may be several genes involved in pollen sterility located on different chromosomes. Intraspecific hybrid pollen sterility reported previously in foxtail millet may also contribute to distorted segregation [29]. In addition, clusters of linked loci experiencing segregation distortion indicated that genetic hitchhiking commonly occurred in this foxtail millet population.

### QTL co-located on chromosome regions

In the present study, many QTL controlling different traits were co-located in the same intervals of the genome. For instance, 8 QTL controlling length, diameter and node number of the main stem; main panicle length and diameter; and straw weight, panicle weight and grain weight per plant were detected in the same interval of Chr. 1. It is a widespread phenomenon in plant genomes that QTL controlling related traits often co-locate in specific intervals. Gupta et al. [21] identified that multi-trait association has been shown by different markers with significant r^2^ value like SSR b129 that is correlated with traits like FLW, PdL, GY, Inf Br, PcL and GW, p75 with GY, GW and PdL. Li et al. [30] indicated that QTL controlling appearance quality in rice were concentrated in a few places, with more than three QTL in the same intervals on Chr. 3, Chr. 5 and Chr. 6. In the present study, co-location of QTL for different traits in the same intervals was consistent with significant positive correlations between these traits. Co-located QTL may be conferred by pleiotropic genes that play important roles in the network of agronomic and yield development of foxtail millet, or by closely-linked alleles from a common parent that confer favorable effects.

### Origin of favorable QTL alleles

In the present study, the two parents had significant differences in agronomic and yield traits. Yugu1 is tall, with less tillering, larger panicles and more grains than Longgu7, reduced seed shattering, and other advantages. Among the 29 QTL identified for 11 agronomic and yield traits, only those controlling panicle neck length had favorable alleles originating from Longgu7. For the other traits, favorable alleles were from Yugu1 as indicated by QTL additive effects, except qLMS6.1. This result confirmed that favorable alleles of traits from the elite Yugu1 are genetically delivered to its progeny, as found in other crops, such as cotton [31–33].

### The potential of QTL for agronomic and yield traits

Stable QTL for agronomic and yield traits are important to functional gene cloning and molecular breeding. To date, the number of QTL identified for agronomic and yield traits is limited in foxtail millet [12, 17–20], and the QTL identified have large confidence intervals and low reliability. Therefore, the QTL mapped to date often fail to meet the requirements of molecular marker assisted selection. Among the 29 QTL identified for 11 agronomic and yield traits in this study, qMPD5.2, and qMPL1.1, and qNNMS7.1 could also be detected in the haplotype map with 0.8 million SNPs [12]. Intergenomic analyses between foxtail millet and sorghum revealed highly conserved collinearity [9]. Comparing QTL in this study with sorghum QTL from a meta-analysis of sorghum QTL trials [34], the region of qLMS6.1 could correspond to the common region of QCL2_7, QCL3_7 and QCL4_7; qMPL2.1 could correspond to QPANLG2.2; qPD3.1 could correspond to QDTFL1_8 and QDTFL2_8; qPD6.1 could correspond to QDTFL1_7 and QDTFL2_7. Additionally, several QTL, such as qPL6.1, qDMS1.1, qSWP1.1, and qPWP1.1 had high additive effects and phenotypic variation explained. QTL that are stable across different populations and species and have high additive effects and phenotypic variation explained are valuable for map-based cloning, candidate gene identification and marker assisted selection.

## Conclusions

A total of 10598 new SSRs were developed and screened to construct a high density intraspecific genetic linkage map for foxtail millet, which included 1035 loci on the nine chromosomes, and spanned 1318.8 cM with 1.27 cM average distance between adjacent markers. A total of 29 QTL were identified for 11 agronomic and yield traits, and the new genetic markers along with genomic-SSRs linked to the QTL may help breeders to construct desirable allelic combinations and accelerate breeding programs for the development of foxtail millet cultivars with improved agronomic performance through MAS.

## Methods

### Development of SSR primers

The reference genome sequence (v2.1) of the foxtail millet genotype ‘Yugu1’ was retrieved from Phytozome (https://phytozome.jgi.doe.gov/pz/portal.html) and simple sequence repeat (SSR) marker primers were designed using SSR locator 1(http://comp.uark.edu/~ashi/MB/SSRLocator.html). The microsatellite motifs were searched by the criteria: eighteen repeat units for mononucleotide (Mono) repeats, nine for dinucleotide (Di) repeats, six for trinucleotide (Tri) repeats, four for tetranucleotide (Tetra) repeats, three for pentanucleotide (Penta) repeats and three for hexanucleotide (Hexa) repeats. The major parameters for designing SSR primers were: (1) primer length from 18 to 27 bases; (2) PCR product size ranges from 100 to 200 bp; (3) melting temperature between 55 and 65 °C with 60 °C being the optimum annealing temperature; (4) GC content of 45–65 % with an optimum of 50 %. SSR primers were named ‘GSA’ and synthesized by Invitrogen Co. Ltd. (Shanghai, China).

### Plant materials and trait examination

Two foxtail millet cultivars, summer cultivated variety Yugu1 and spring cultivated variety Longgu7, were chosen as parents for the mapping population. Yugu1 is characterized by a long growth period, tall plant height, minimal tillering, large panicle, many grains and minimal seed shattering. Longgu7 is characterized by extreme early maturity, short plant height and a small panicle. The mapping parents were crossed in winter of 2012 in Sanya, Hainan, China. F_1_ individuals were self-pollinated to produce F_2_ seeds at Sanya, Hainan, during spring 2013. Parents and 167 F_2_ plants were planted in Tianshui, Gansu, during summer 2013. One hundred sixty-seven F_2_-derived lines were self-pollinated to produce F_2:3_ and F_2:4_ lines. Agronomic and yield traits were evaluated for F_2_ individuals planted in summer 2013 and F_2:4_ lines planted in summer 2014 in Tianshui, Gansu. Data were collected on period of duration (PD, d), peduncle length (PL, cm), length of main stem (LMS, cm), diameter of main stem (DMS, cm), node number of the main stem (NNMS, no), main panicle length (MPP, cm), main panicle diameter (MPD, cm), straw weight per plant (SWP, g), panicle weight per plant (PWP, g), grain weight per plant (GWP, g), and 1000-grain weight (TGW, g). For F_2:3_ lines planted in Sanya, Hainan, 167 F_2:3_ family lines were sowed in November 2013, and harvested in January 2014. During this period, the plants grew weakly under low temperature and trait data were not measured.

### SSR marker assays

Total genomic DNA from fresh young leaves of the parents and 167 F_2_ individuals were extracted according to a modified CTAB method [35]. All newly developed SSR primer pairs were screened for polymorphism between the mapping parents and those showing clear polymorphism were used to genotype the F_2_ population. PCR amplification and product testing were performed according to Zhang [35]. Clear polymorphic DNA bands on the gels were used for scoring and genotyping. Loci detected were named with the primer name. For multiple polymorphic loci revealed by the same primer pair, an extra letter was added to the primer name, such as a/b/c, indicating the molecular size from the smallest to the largest.

### Genetic map construction

The segregation of each SSR marker was tested by a Chi-squared test to determine if it deviated significantly from the expected Mendelian segregation ratio. JoinMap 4.0 [36] was used to group and order all loci with a LOD threshold of 5.0. The Kosambi mapping function was used to convert recombination frequencies into map distances [37].

### QTL mapping

The multiple QTL mapping method of MapQTL 6.0 [38] was implemented to identify QTL and estimate their effects. LOD ≥2.5 was used to declare suggestive QTL. Positive additive effects of QTL indicated that the Yugu1 allele increased the phenotypic value, whereas negative effects indicated that the Longgu7 allele increased the phenotypic value. QTL names started with ‘q’, followed by a trait abbreviation (e.g. PD for period) and the chromosome number, followed by the number of QTL controlling the same trait on the chromosome. Graphical representation of the genetic map and QTL bars representing 1-LOD reduction in likelihood was carried out with Map Chart 2.2 [39].

### Ethical standard

The authors note that this research was performed and reported in accordance with ethical standards of scientific conduct.

## Ethics approval and consent to participate

Not application.

## Consent for publication

Not applicable.

## Availability of data and material

The premier data designed in this publication have been deposited in the probe databases of NCBI in http://www.ncbi.nlm.nih.gov/probe/?term=JAK%5Bsubm%5D%20. The data sets supporting the results of this article are included within the article and its additional files.

## Additional files

Additional file 1: Table S1. Phenotypic data analysis of 11 agronomic traits and yield traits for 167 F2 progeny. (XLSX 10 kb) Additional file 2: Figure S1. Phenotypic frequency distribution of 11 agronomic and yield traits in Yugu1 × Longgu7 F2 families. (DOC 143 kb) Additional file 3: Table S2. Correlation coefficients among agronomic and quality traits in 167 F2 progeny in 2013 and 2014. (XLSX 11 kb) Additional file 4: Table S3. Newly SSR markers developed from the released ‘Yugu1’ genomic sequence. (XLS 2505 kb) Additional file 5: Figure S2. Biased distributions of SSR motifs. (DOC 281 kb)

## Abbreviations

EST: expressed sequence tag.
PIC: polymorphic information content
QTL: quantitative trait locus
SSR: simple sequence repeat
