# Supplementary material for: A high density genetic map and QTL for agronomic and yield traits in Foxtail millet [Setaria italica (L.) P. Beauv.]
Source: BMC Genomics. 2016 May 4;17:336. doi: 10.1186/s12864-016-2628-z (PMC4857278; doi:10.1186/s12864-016-2628-z)
Supplement: Additional file 2: Figure S1. — Phenotypic frequency distribution of 11 agronomic and yield traits in Yugu1 × Longgu7 F2 families. (DOC 143 kb) [file 12864_2016_2628_MOESM2_ESM.doc]

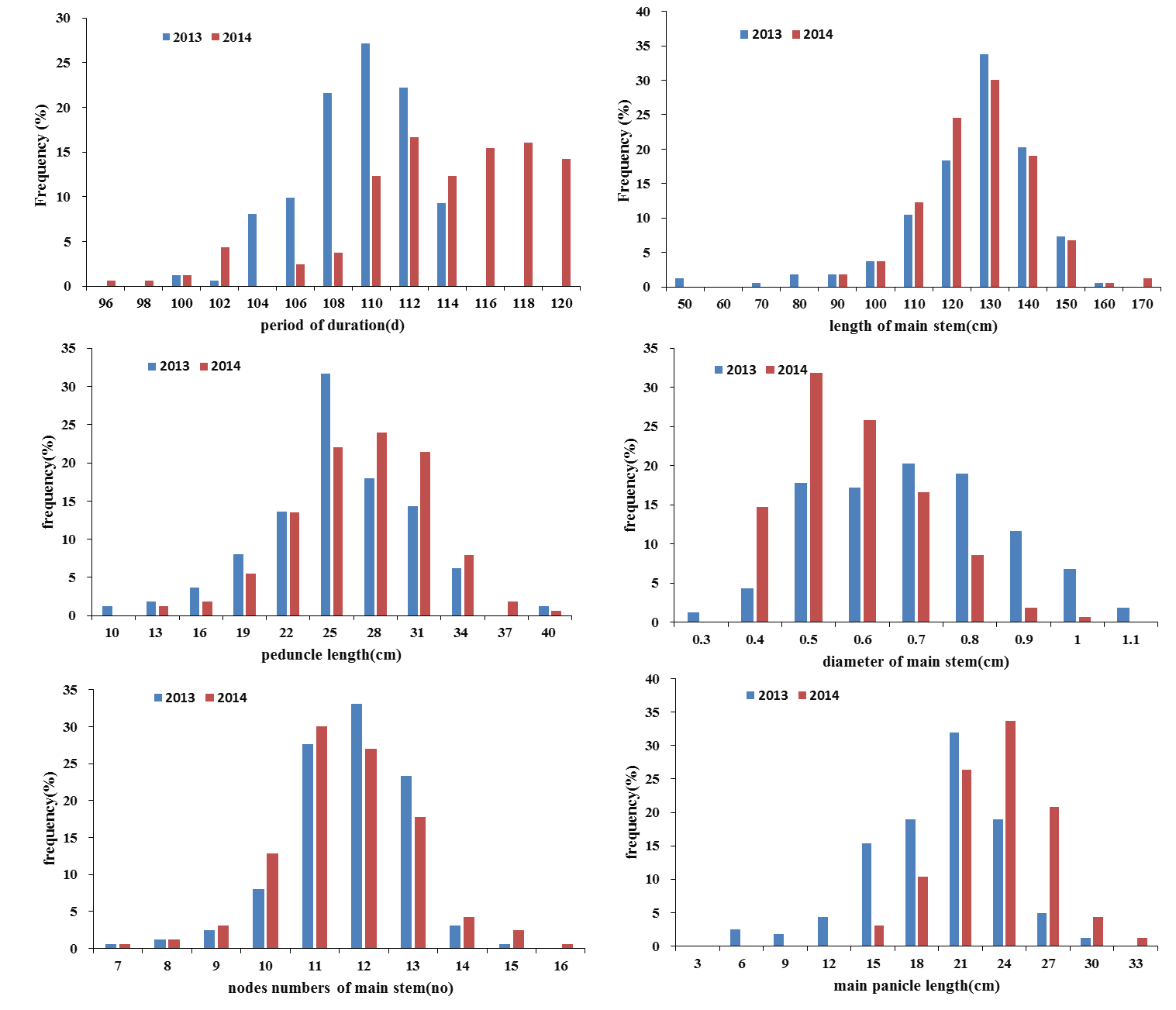

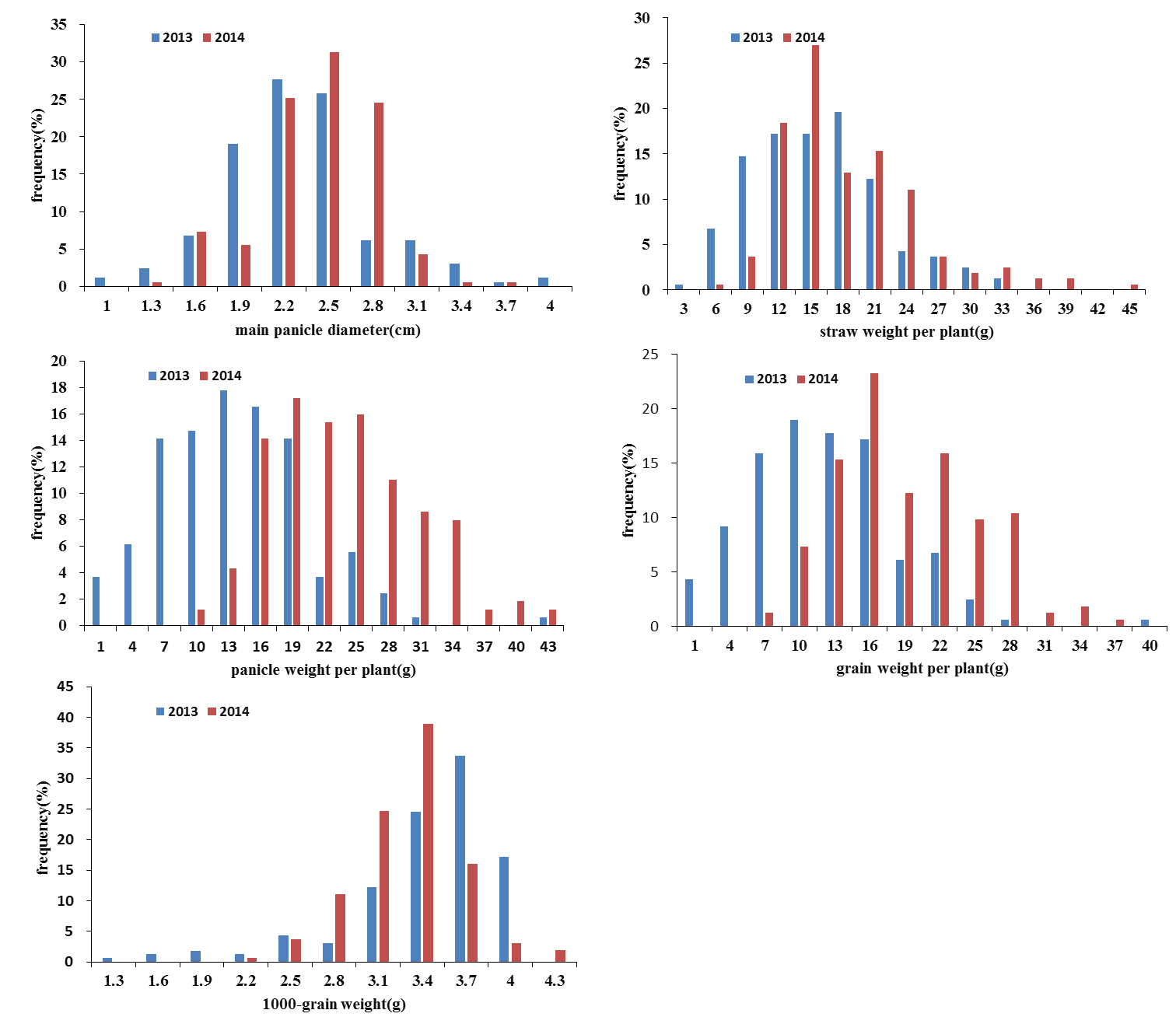


Figure S1 Phenotypic frequency distribution of 11 agronomic traits and yield traits in (Yugu1 × Longgu7) F_2_ individuals and F_2:4_ family lines
