# Supplementary figures and images for: A high density genetic map and QTL for agronomic and yield traits in Foxtail millet [Setaria italica (L.) P. Beauv.]
Source: BMC Genomics. 2016 May 4;17:336. doi: 10.1186/s12864-016-2628-z (PMC4857278; doi:10.1186/s12864-016-2628-z)

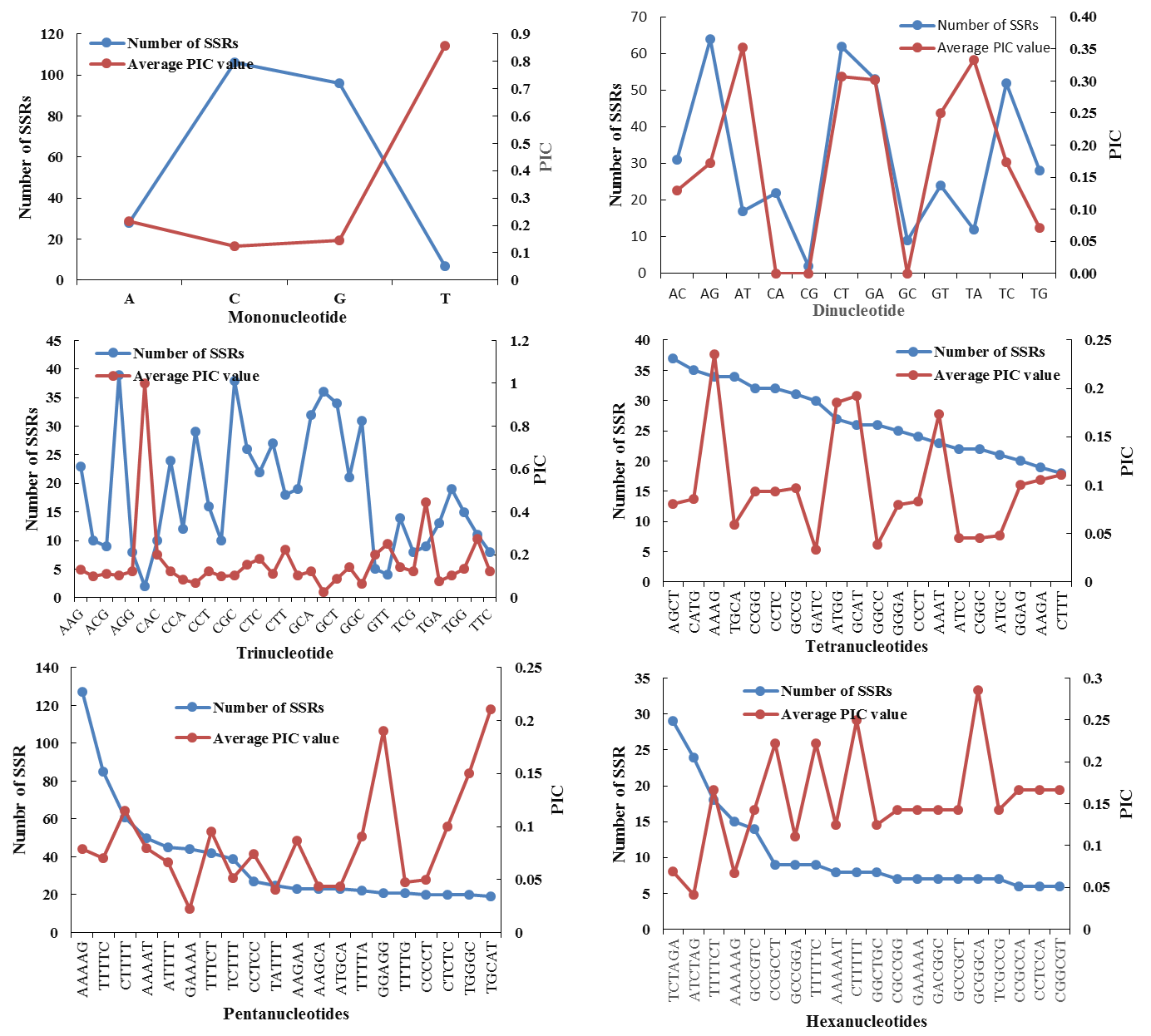


Figure S2 Biased distributions of the amount of each kind of SSR motifs

Supplement: Additional file 5: Figure S2. — Biased distributions of SSR motifs. (DOC 281 kb) [file 12864_2016_2628_MOESM5_ESM.doc]
